# Pore Structure of Grain-Size Fractal Granular Material

**DOI:** 10.3390/ma12132053

**Published:** 2019-06-26

**Authors:** Yifei Liu, Dong-Sheng Jeng

**Affiliations:** 1 School of Civil Engineering, Southwest Jiaotong University, Chengdu 610031, China; yfliu@my.swjtu.edu.cn; 2 School of Engineering & Built Environments, Griffith University Gold Coast Campus, Gold Coast, QLD 4222, Australia

**Keywords:** grain-size fractal, particle packing, pore structure, pore size distribution

## Abstract

Numerous studies have proven that natural particle-packed granular materials, such as soil and rock, are consistent with the grain-size fractal rule. The majority of existing studies have regarded these materials as ideal fractal structures, while few have viewed them as particle-packed materials to study the pore structure. In this study, theoretical analysis, the discrete element method, and digital image processing were used to explore the general rules of the pore structures of grain-size fractal granular materials. The relationship between the porosity and grain-size fractal dimension was determined based on bi-dispersed packing and the geometric packing theory. The pore structure of the grain-size fractal granular material was proven to differ from the ideal fractal structure, such as the Menger sponge. The empirical relationships among the box-counting dimension, lacunarity, succolarity, grain-size fractal dimension, and porosity were provided. A new segmentation method for the pore structure was proposed. Moreover, a general function of the pore size distribution was developed based on the segmentation results, which was verified by the soil-water characteristic curves from the experimental database.

## 1. Introduction

In natural environments, various types of materials are formed by grain packing. For example, soil is directly generated by particle packing, while rock is formed by siliceous or calcareous cementation on the basis of sediment packing. Concrete is cemented with cement according to coarse aggregate packing. For such granular materials, let *r* denote the particle size and N(≥r) denote the number of particles larger than *r*. The grain size distribution satisfies the grain-size fractal rule if the following relationship exists:

(1) $$N\left(\geq r\right)\propto r^{-D},$$

where *D* is the number-size distribution fractal dimension (also referred as grain-size fractal dimension in this research).

It is well known that fractals play an important role in the natural environment [1]. Numerous studies have reported that soil is a grain-size fractal granular material [2,3,4,5,6]. The grain-size distribution of sedimentary rock has been found to follow power-laws with a fractal dimension in the range of 2 to 3 orders [7,8]. Fragments of a geological body produced by weathering, explosions, and impacts often satisfy a grain-size fractal distribution over a wide range of scales, owing to the scale invariant of the fragmentation mechanism [9,10,11,12,13]. Furthermore, in engineering practice, numerous granular materials are generated by rock fragment packing, such as ore deposits and concrete coarse aggregates, which also follow the grain-size fractal distribution [14]. Therefore, it is important to understand the general properties of grain-size fractal granular materials.

Similar to most basic characteristics, the pore structure determines various physical and mechanical properties of granular materials. The soil-water characteristic curve is mainly determined by the soil pore structure [15]. Therefore, during the early research stages, the pore distribution coefficient was commonly used to represent the effect of the pore structure [16,17,18]. Furthermore, the hydraulic conductivity of granular media is mainly dependent on their pore structure. Numerous studies have been conducted on the prediction of the permeability based on the pore size distribution available in the literature [19,20,21]. The filtration performance of a granular medium is also determined by its pore structure, particularly the constriction distribution [22,23,24]. For suffusion and internal erosion, pore structure also plays a decisive role [25,26]. The pore structure holds the same significance in various other fields, such as shale gas engineering [27], petroleum engineering [28,29], and pollutant transport and reaction in environmental engineering [30,31,32].

At present, numerous studies regarding the pore structure of granular media are available in the literature. Several researchers regard the granular media formed by particle packing as ideal fractal structures, such as Menger sponges [33,34,35,36,37,38]. In these works, the permeability or soil-water characteristics have been studied based on the ideal fractal structures. In reality, the ideal fractal structure is quite different from the grain-size fractal granular media formed by particle packing, because the physical packing process is not considered. Numerous researchers have extracted meso-scopic images of granular media by means of computed tomography (CT) or nuclear magnetic resonance (NMR), and then studied their pore structure using digital image processing technology [39,40,41,42,43,44]. Existing studies on the pore structure based on CT and NMR could only examine several rock or soil samples simultaneously, and were therefore unable to obtain general rules. Certain studies have used the discrete element method (DEM) to generate granular media and investigate their pore structure [24,45,46,47,48,49,50]. These DEM studies focused on the pore structures of mono-sized packing, bi-dispersed packing, and packing with different gradation parameters. To date, except the simple 2D pore-structure studies [51,52], few studies regarding grain-size fractal granular material based on particle packing are available in the literature. Furthermore, the aforementioned DEM studies only presented the pore size distribution curves for several specific cases, and no general law was provided.

The digital image processing approaches used in pore structure studies can be categorized into Delaunay tessellation, medial axis, and watershed-based methods. Each of these approaches exhibits limitations. For example, the Delaunay tessellation-based method [24,45,48,50] can only be applied to spherical particles, and when the number of particles around the pore is greater than four, the results become inaccurate. In the medial axis-based methods [43,44,53,54,55,56], the medial axis of the pore structure needs to be extracted first. For a complex pore structure, particularly with a large protuberance on the pore surface, the extraction of the medial axis is generally inaccurate. In watershed-based methods [42,57], the pore structure first needs to be converted into the Euclidean distance field, which has a relatively large computational cost. Furthermore, over-segmentation of the pore structure often occurs when the pore boundaries are complex. In the aforementioned studies, the identification criteria of the throat and pore are not uniform, and neither are the merging criteria of the pore. Different criteria will lead to varying results [58,59,60], therefore, it is difficult to establish a general rule for pore distribution according to the above methods.

The objective of this study is to investigate the general packing rule and pore structure characteristics of grain-size fractal granular material. By means of theoretical analysis and DEM simulation of the packing process, the relationship between the porosity and number-size distribution fractal dimension was established. Based on the DEM packing pattern, the pore structures of granular materials with different fractal dimensions and compactness were extracted. Thereafter, the fractal features of the pore structure, such as the box-counting dimension, lacunarity, and succolarity, were studied. Moreover, a new method for pore segmentation was proposed, based on the continuous open operation. A general function of the pore size distribution was also obtained in this study according to the segmentation results. Finally, the pore size distribution function was verified by the soil-water characteristic curve.

## 2. Porosity

### 2.1. Theoretical Analysis

As one of basic parameters of granular materials, the porosity is determined by the particle size distribution. A certain relationship must exist between the porosity and grain-size fractal dimension (*D*), which essentially represents the particle size distribution. In this study, a formula for porosity prediction was derived based on the concept of integrating discrete bi-dispersed packing and discrete geometric packing, as proposed by Brouwers [61].

The basic idea to derive the porosity prediction formula can be summarized as follows. There is exact solution of ideal geometric packing. At the same time, the porosity of bi-dispersed packing are easy to be obtained by simulation or experiment. The exact solution of geometric packing is modified to a general form by the results of bi-dispersed packing, which is applicable to real particle packing. According to the particularity of grain-size fractal, the final porosity prediction equation is obtained by modifying the general form.

#### 2.1.1. Bi-Dispersed Packing

Bi-dispersed packing refers to the packing of two different sized particles. Supposing that the radius of large particle is $d_{l}$ and that of the small particle is $d_{s}$, the volume fraction of the large particle is $c_{l}$, while that of the small particle is $c_{s}$. Then, the size ratio $d_{r}$ is defined as

(2) $$d_{r}=\frac{d_{l}}{d_{s}},$$

and the volume fraction ratio $c_{r}$ is defined as

(3) $$c_{r}=\frac{c_{l}}{c_{s}}.$$

The porosity of bi-dispersed packing is dependent on the size ratio $d_{r}$ and volume fraction ratio $c_{r}$.

**Figure 1:**
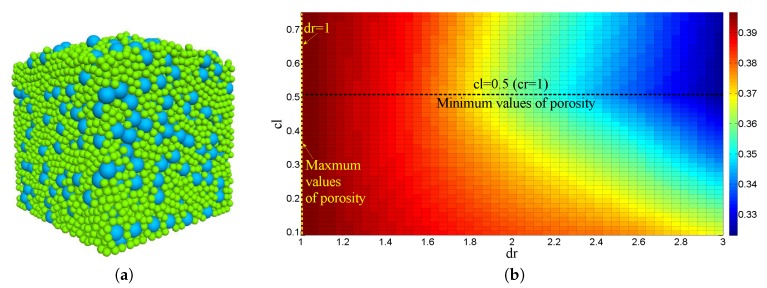
Schematic of (**a**) bi-dispersed packing, and (**b**) its porosity distribution with radius ratio $d_{r}$ and volume fraction ratio $c_{r}$.

In this study, the DEM was used to simulate the packing process of a bi-dispersed particle system with different size ratios $d_{r}$ and volume fraction ratios $c_{r}$ under the action of gravity. The packing schematic is presented in Figure 1a. The porosity obtained by the DEM simulation is illustrated in Figure 1b. It can be observed that, as the particle size ratio $d_{r}$ decreases, the porosity increases, and maximum values occur at $d_{r}=1$. Meanwhile, with the increase in the volume fraction ratio $c_{r}$, the porosity first decreases and then increases, and minimum values occur when $c_{r}=1$. The variation in the porosity is consistent with the conclusions of Furnas [62]. The porosity ϕ of the bi-dispersed packing can be expressed as a function of $d_{r}$ and $c_{r}$:

(4) $$\phi =f_{p}\left(d_{r},c_{r}\right).$$

As there are maximum values of ϕ at $d_{r}=1$ and minimum values at $c_{r}=1$, the following relationship can be obtained:

(5) ∂fp∂cr|dr=1,cr=1=0.

#### 2.1.2. Geometric Packing

**Figure 2:**
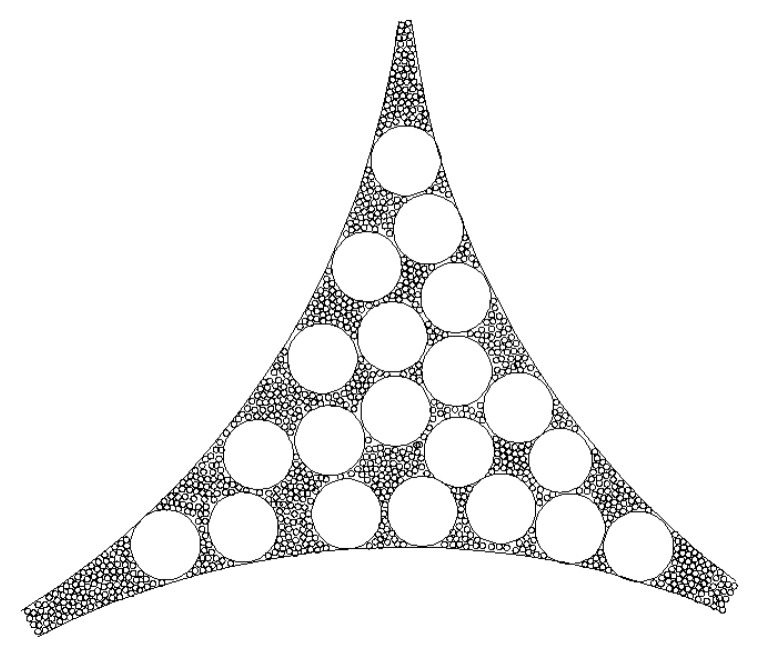
Schematic of geometric packing (taking 2D as an example).

As illustrated in Figure 2, geometric packing refers to the packing of large particles, followed by the filling of small particles in the pores of the large particle packing. During the filling of small particles, only the pores formed by the skeleton of the large particles were filled, which had no effect on the packing of the large particles.

To satisfy the geometric packing condition, the size ratio of the two adjacent groups ($d_{i}$ and $d_{i+1}$) needs to be greater than a certain value, i.e.,

(6) $$\frac{d_{i}}{d_{i+1}}=d_{r}>d_{\mathrm{rt}},$$

in which the threshold value $d_{\mathrm{rt}}$ is in the range of approximately 7 to 10 according to the study of Furnas [62].

As no interaction between large and small particles occurs in the packing process, the porosity of geometric packing can be strictly derived. Assume that the geometric packing consists of *n* groups of particles, in which the maximum particle size is $d_{1}$ and the minimum particle size is $d_{n}$. Firstly, the largest particles are stacked. At this time, the volume fraction of the particles is set to $c_{1}$ and the pore volume fraction can be expressed as

(7) $$\phi_{1}=1-c_{1}.$$

Then, the second group of particles is filled in the pores generated by the packing of the first group. At this time, the volume fraction of the particles of the second group is

(8) $$c_{2}=c_{1}\phi_{1}=c_{1}\left(1-c_{1}\right).$$

After the second group is filled, the total volume fraction of the particles $c_{t2}$ and pores $\phi_{2}$ can be expressed as follows:

(9) $$c_{t2}=c_{1}+c_{2}=c_{1}+c_{1}\left(1-c_{1}\right),$$

(10) $$\phi_{2}=1-c_{t2}={\left(1-c_{1}\right)}^{2}=\phi_{1}^{2}.$$

By analogy, it can be concluded that, when *n* groups of particles are stacked, the total volume fractions of the particles $c_{tn}$ and pores $\phi_{n}$ are

(11) $$c_{tn}=c_{1}+c_{1}\left(1-c_{1}\right)+c_{1}{\left(1-c_{1}\right)}^{2}+...+c_{1}{\left(1-c_{1}\right)}^{n-1},$$

(12) $$\phi_{n}=1-c_{tn}={\left(1-c_{1}\right)}^{n}=\phi_{1}^{n}.$$

When the size ratio of the two adjacent groups is smaller than the threshold value ($d_{r}<d_{\mathrm{rt}}$), the large and small particles will interact with one another, and the formula derived previously is no longer valid. To obtain the porosity prediction formula in the case of $d_{r}<d_{\mathrm{rt}}$, Equation (4), obtained by bi-dispersed packing, is introduced into (12), i.e., when the second group of particles is stacked based on the packing of the first group, $\phi /\phi_{1}$ is used to multiply the porosity of the first group packing ($\phi_{1}$) instead of $\phi_{1}$. By analogy, the final porosity after the packing of *n* groups of particles is

(13) $$\phi_{n}=\phi_{1}{\left(\frac{\phi }{\phi_{1}}\right)}^{n-1}=\phi_{1}{\left(\frac{f_{p}\left(d_{r},c_{r}\right)}{\phi_{1}}\right)}^{n-1}.$$

When $d_{r}>d_{\mathrm{rt}}$, $\phi =\phi_{1}^{2}$. At this time, (13) is reduced to (12). Therefore, (13) is applicable to all cases of the size ratio $d_{r}$ as a general formula.

#### 2.1.3. Packing of Grain-Size Fractal Granular Material

For grain-size fractal granular media, if the maximum particle size $d_{\max}$ is known, (1) can be changed into the following form [35]:

(14) $$N\left(\geq d\right)={\left(\frac{d_{\max}}{d}\right)}^{D}.$$

The total number of particles can be obtained by (14), which is

(15) $$N_{t}={\left(\frac{d_{\max}}{d_{\min}}\right)}^{D},$$

where $d_{\min}$ denotes the minimum particle size. The differential form of (14) is

(16) $$-dN\left(d\right)=Dd_{\max}^{D}d^{-(D+1)}dd.$$

According to (15) and (16), we can obtain

(17) $$-dN\left(d\right)/N_{t}=Dd_{\min}^{D}d^{-(D+1)}dd=f_{f}\left(d\right)dd.$$

From (17), the probability density function for any particle size in grain-size fractal granular material is provided:

(18) $$f_{f}\left(d\right)=Dd_{\min}^{D}d^{-(D+1)}.$$

It is assumed that continuous fractal porous media are divided into *n* groups of particles with the same size ratio of two adjacent groups (when n→∞, the particle size distribution tends to be continuous), i.e.,

(19) $$\frac{}{}d_{i}/d_{i+1}=d_{r}.$$

Meanwhile, let the maximum particle size be $d_{1}$, and the minimum particle size be $d_{n}$. From (19), we can obtain

(20) $$\frac{d_{\max}}{d_{\min}}=\frac{d_{1}}{d_{n}}=d_{r}^{n}.$$

Let $A=d_{\max}/d_{\min}$. Then, the size ratio $d_{r}$ can be expressed as follows, according to (20) and the Taylor expansion:

(21) $$d_{r}=A^{\frac{1}{n}}=1+\frac{1}{n}\ln A+O\left(\frac{1}{n^{2}}\right).$$

**Figure 3:**
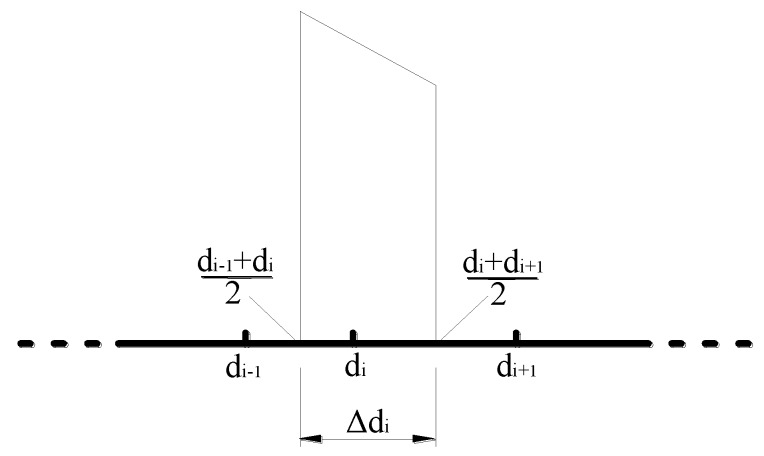
Schematic of distribution width of *i*th group.

Thereafter, the volume fraction $c_{i}$ of the *i*th group is studied. As illustrated in Figure 3, based on (19), the distribution width of the *i*th group can be expressed as

(22) $$\Delta d_{i}=\frac{d_{i-1}+d_{i}}{2}-\frac{d_{i}+d_{i+1}}{2}=\frac{d_{r}-\frac{1}{d_{r}}}{2}d_{i}.$$

Let the total volume fraction of particles be $F_{\mathrm{vt}}$, and the volume of a single particle be $Bd_{i}^{3}$ (*B* is the shape factor). According to (18) and (22), the volume fraction $c_{i}$ can be obtained as follows:

(23) $$c_{i}=\frac{f_{f}\left(d_{i}\right)\Delta d_{i}Bd_{i}^{3}}{F_{\mathrm{vt}}}=\frac{1}{F_{\mathrm{vt}}}Dd_{\min}^{D}\frac{d_{r}-\frac{1}{d_{r}}}{2}Bd_{i}^{3-D}.$$

From (23), the volume fraction ratio of two adjacent groups is provided:

(24) $$c_{ri}=\frac{c_{i}}{c_{i+1}}={\left(\frac{d_{i}}{d_{i+1}}\right)}^{3-D}.$$

As can be observed in (24), the volume fraction ratio $c_{ri}$ does not vary with the groups, and is only related to the size ratio and fractal dimension. Thus, $c_{r}$ is used instead of $c_{ri}$ in the following. The Taylor expansion is applied to (24) at $d_{r}=1$, and the volume fraction ratio can be expressed as

(25) $$c_{r}=d_{r}^{3-D}=1+\left(3-D\right)\left(d_{r}-1\right)+O{\left(d_{r}-1\right)}^{2}.$$

Substituting (21) into (25) yields

(26) $$c_{r}=1+\frac{3-D}{n}\ln A+O\left(\frac{1}{n^{2}}\right).$$

At this time, the size ratio ($d_{r}$) and volume fraction ratio ($c_{r}$) of the adjacent groups in the grain-size fractal granular material are expressed as functions of the number of groups (*n*), fractal dimension (*D*), and size ratios of the maximum and minimum particles (*A*), respectively, as indicated in (21) and (26).

**Figure 4:**
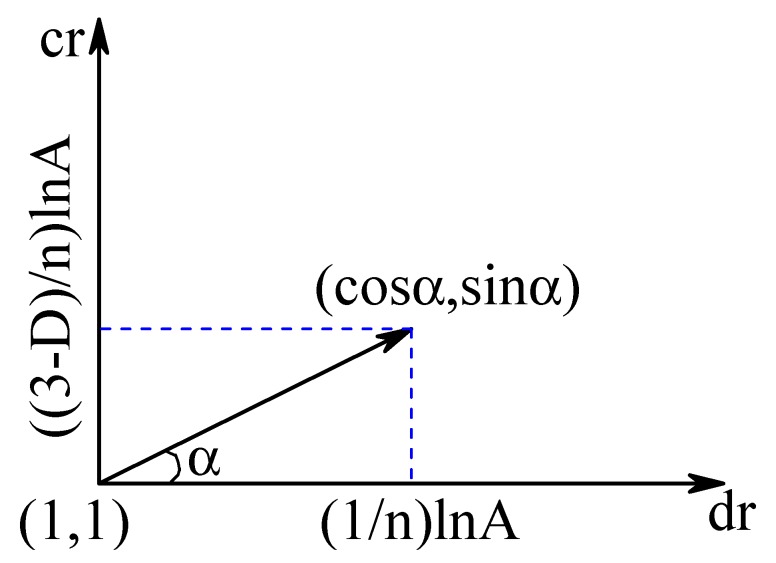
Direction of Taylor expansion.

Continuous grain-size fractal granular material refers to the case in which the number of groups n→∞, which implies that the size ratio $d_{r}\to 1$ and the volume fraction ratio $c_{r}\to 1$. Based on (21) and (26), the Taylor expansion of (4) is applied near $d_{r}=1$ and $c_{r}=1$ along the direction indicated in Figure 4, which can be expressed as

(27) fp(dr,cr)|dr→1,cr→1=fp(1+1nlnA+O(1n2),1+3−DnlnA+O(1n2))=fp(1,1)+cosαnlnA∂fp∂dr|dr=1,cr=1+sinα(3−D)nlnA∂fp∂cr|dr=1,cr=1+O(1n2).

According to Figure 4, when $\Delta d_{r}$ takes $\frac{1}{n}\ln A$, the corresponding $\Delta c_{r}$ takes $\frac{3-D}{n}\ln A$, so that a certain relationship of α exists:

(28) $$\cos\alpha =\frac{1}{1+{\left(3-D\right)}^{2}}.$$

Let $\beta =\frac{\partial f_{p}}{\partial d_{r}}{|}_{d_{r}=1,c_{r}=1}$, and the porosity of the mono-sized packing is $\phi_{1}$ (i.e., $f_{p}\left(1,1\right)=\phi_{1}$). Neglecting the high-order small term ($O(\frac{1}{n^{2}})$), and substituting (5) and (28) into (27) yields

(29) fp(dr,cr)|dr→1,cr→1=ϕ1+β(1+(3−D)2)1nlnA.

When n→∞, the discrete grain-size fractal granular material is converted into a continuous form. After substituting (29) into (13), the final porosity of the grain-size fractal packing can be obtained by calculating the limit as follows:

(30) $$\begin{array}{c} \phi =\lim_{n\to \infty }\phi_{1}{\left(\frac{f_{p}\left(d_{r},c_{r}\right)}{\phi_{1}}\right)}^{n-1}=\lim_{n\to \infty }\phi_{1}{\left(1+\frac{\beta }{\phi_{1}\left(1+{\left(3-D\right)}^{2}\right)}\frac{1}{n}\ln A\right)}^{n-1}\\ =\phi_{1}A^{\frac{\beta }{\phi_{1}\left(1+{\left(3-D\right)}^{2}\right)}}. \end{array}$$

As indicated in (30), the porosity of the grain-size fractal granular material is expressed by a function of the porosity of the mono-sized packing ($\phi_{1}$), fractal dimension (*D*), size ratio of the maximum and minimum particles (*A*), and porosity partial derivative of the bi-dispersed packing at $d_{r}=1$ and $c_{r}=1$ (β). Equation (30) is a semi-empirical formula, in which the values of $\phi_{1}$ and β can be obtained by DEM simulation, as discussed in the following section.

### 2.2. Numerical Simulation

The DEM proposed by Cundall and Strack [63] was used to simulate the packing process of particles under the action of gravity. Although there are some new packaging algorithms which are more efficient and flexible [64], DEM is still the closest algorithm to the real gravity packing process. In this study, the DEM was implemented by the software of PFC${}^{3D}$. The linear elastic model of PFC${}^{3D}$ was used for the interactions among particles. When the elastic modulus is large, the deformation of particles is very small, and the effect on the porosity can be ignored. Newton’s second law is used in the DEM to describe the movement of particles, which is the same as the packing process in reality.

The friction coefficient of the particles has a significant influence on the packing results [65]. A larger friction coefficient results in a faster kinetic energy loss, which leads to looser packing of particles. In soil mechanics, the relative density is used to represent the soil compactness. According to the study of Huang et al. [65], a certain relationship exists between the friction coefficient and relative density when the DEM is used to simulate particle packing. Therefore, granular material with different compactness values can be produced by changing the friction coefficient $\mu_{p}$ of the particles.

**Figure 5:**
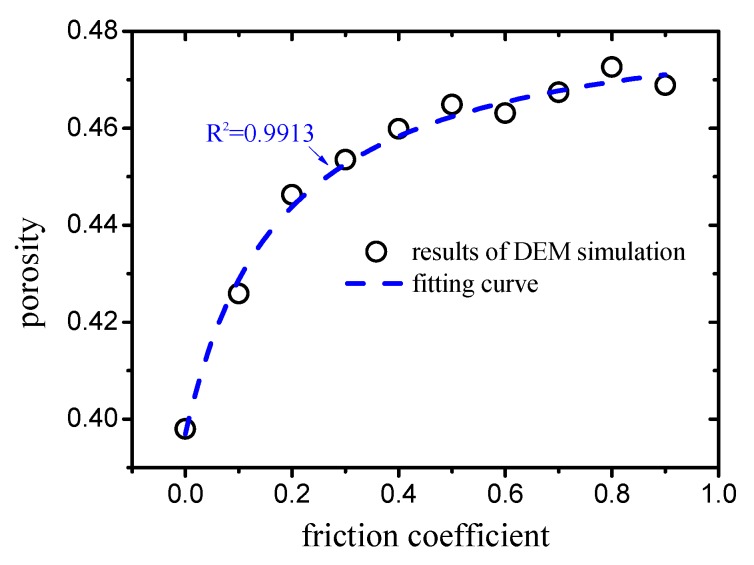
Relation between friction coefficient and porosity of mono-sized packing.

In (30), the parameters $\phi_{1}$ and β of the granular material differ under varying compactness conditions. As illustrated in Figure 5, the porosity $\phi_{1}$ of mono-sized packing with different compactness values can be obtained by DEM simulation with different particle friction coefficients. It can be observed from the figure that, with an increase in the friction coefficient, the porosity increases nonlinearly. A fitting formula with a strong fitting degree ($R^{2}=0.9913$) was provided, which is expressed as:

(31) $$\phi_{1}=\arctan\left(63.0\mu_{p}+11.2\right)-1.1.$$

**Figure 6:**
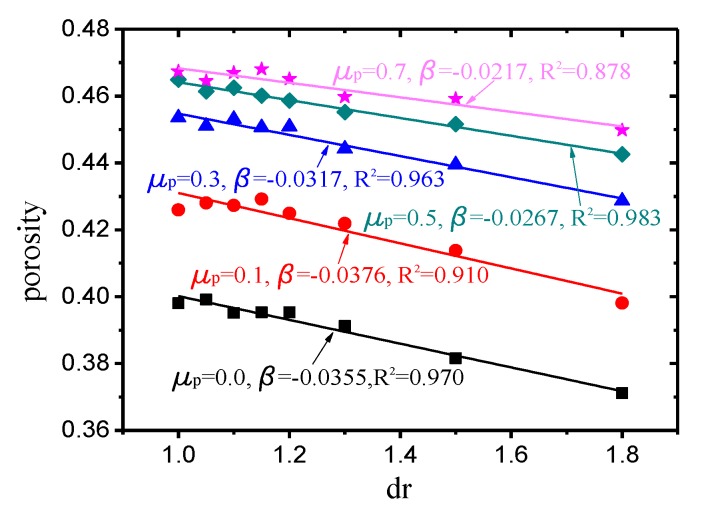
β values of different friction coefficients obtained by bi-dispersed packing.

The parameter β was defined as $\beta =\frac{\partial f_{p}}{\partial d_{r}}{|}_{d_{r}=1,c_{r}=1}$, which indicates the value of the porosity partial derivative with respect to the size ratio ($d_{r}$) at $d_{r}=1$ and $c_{r}=1$ under bi-dispersed packing. For each compactness value, the porosity of several discrete points at $c_{r}=1$ and $d_{r}\to 1$ was calculated by simulating the bi-dispersed packing. As illustrated in Figure 6, the slope obtained by linear regression of the calculated porosity was the β value corresponding to each friction coefficient (compactness). Note here that these two parameters are obtained by PFC${}^{3D}$ which is spherical DEM. In practice, because the shape of the particles is not spherical usually, the coefficient of friction of the particles is different, etc., $\phi_{1}$ and β needs to be given by experiments.

After obtaining the values of $\phi_{1}$ and β under the conditions of different friction coefficients, and substituting these into (30), the porosity corresponding to different fractal dimensions could be predicted by (30). To verify (30), the packing process of the grain-size fractal granular material with different fractal dimensions and compactness values was simulated by means of the DEM.

**Figure 7:**
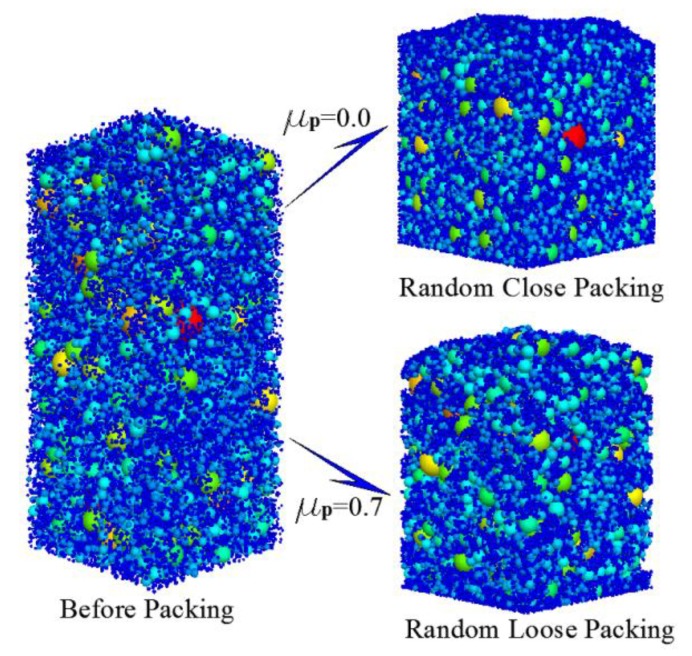
Packing process of grain-size fractal granular material (taking *D* = 2.7 as an example).

**Figure 8:**
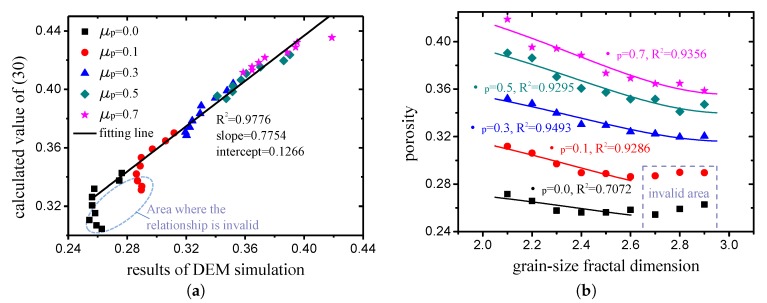
(**a**) Comparison of porosity measured from DEM simulation with values calculated by (30); (**b**) porosity of grain-size fractal granular media with different fractal dimensions and friction coefficients (the scattered points represent the results of the DEM simulation, while the solid lines represent the results calculated by (30)).

The simulation of the packing process can be divided into three steps. Firstly, according to the probability density function (18) and total number of particles, the numbers of each particle size are calculated. Secondly, as illustrated in Figure 7, particles with different diameters are randomly distributed in the space. Finally, the randomly distributed particles are stacked under the action of gravity until the packing is stable, following which the porosity is calculated. The simulation results are presented in Figure 8.

The sphere packing problem has attracted the interest of mathematicians and physicists for many centuries, and great names, such as Kepler, Newton, and Descartes, are associated with this problem. A jamming phenomenon occurs in hard-particle packing. Owing to the uncertainty of the jammed structure, it is difficult to obtain an exact solution for the porosity, even for mono-sized particle packing. Jammed bi-dispersed packing and multi-sized packing have received a certain amount of attention, but their characterization is far from complete. It is impossible to precisely predict the porosity of multi-sized particle packing using the currently available techniques [66,67].

As can be observed in Figure 8a, a linear correlation exists between the results of the DEM simulation and calculated values of (30). This demonstrates that (30) can capture the rule of the grain-size fractal packing to a significant extent. Meanwhile, if $\phi_{1}$ and β are used as fitting parameters, and (30) is used to fit the porosity obtained by the DEM simulation, excellent fitting degrees appear, as indicated in Figure 8b. It can also be observed from Figure 8 that the relationship (30) is invalid in the area where the random packing is close ($\mu_{p}<0.3$) and the fractal dimension is large (D>2.6). This is because tighter packing results in a more obvious jamming phenomenon, which implies that the theoretical state of the densest packing cannot be achieved. For the region in which the relationship is invalid ($\mu_{p}<0.3$, D>2.6), the porosity does not continue to decrease as the fractal dimension increases, so the porosity of D=2.6 can be regarded as the porosity of the cases when D>2.6. In summary, the relationship between the porosity and fractal dimension of the grain-size fractal granular material was established by means of theoretical analysis and DEM simulation, which can be used to predict the porosity.

## 3. Geometric Characteristics of Pore Structure

**Figure 9:**
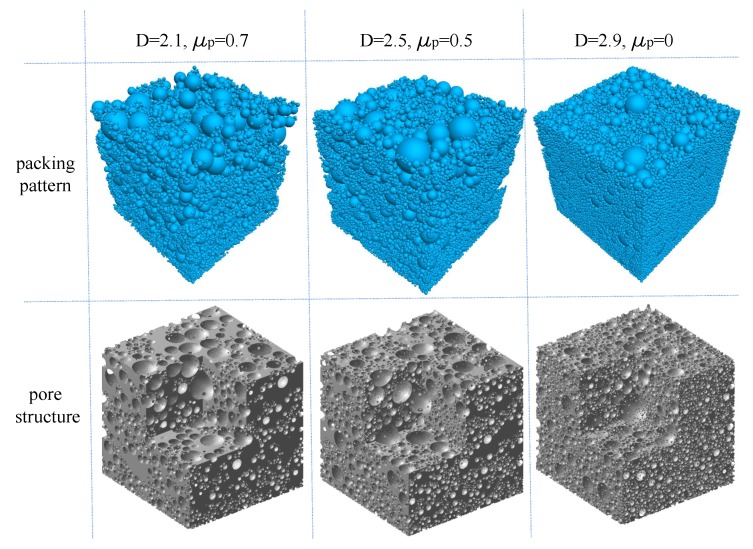
Packing patterns and pore structures of three typical cases.

Based on the packing results presented in Section 2.2, the pore structure of each grain-size fractal granular material was extracted by MATLAB programming for geometric feature analysis. Taking three cases (D=2.1, $\mu_{p}=0.7$; D=2.5, $\mu_{p}=0.5$; and D=2.9, $\mu_{p}=0.0$) as examples, the final pattern of the packing particles and extracted pore structure are illustrated in Figure 9. In this study, the voxel matrix size of the pore structure was 512×512×512. Then, based on the three-dimensional (3D) binary image of the pore structure, digital image processing technology was applied to study the fractal properties and pore size distribution.

### 3.1. Fractal Properties

#### 3.1.1. Box-Counting Dimension

The box-counting dimension, also known as the Minkowski dimension, is the natural structural property of a fractal object, representing the amount of measurement space occupied by the object, i.e., the object complexity (fragmentation degree). The 3D box-counting fractal dimension $D_{\mathrm{bc}}$ used in this study can be described as follows [1]:

(32) $$\ln N_{b}=D_{\mathrm{bc}}\ln\left(\frac{1}{r_{b}}\right)+\ln k,$$

where $N_{b}$ is the number of boxes covering the pore space, $r_{b}$ is the side of a box, and *k* is a constant which has no effect on the box-counting dimension. Moreover, $D_{\mathrm{bc}}$ is the slope of the linear part within the cutoff lengths in the log-log plot.

**Figure 10:**
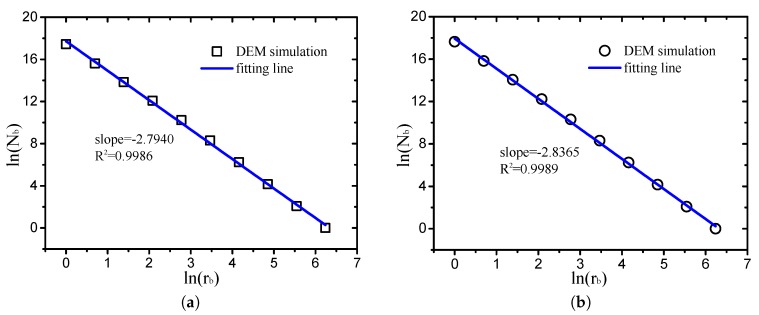
Log-log plots of box size and number of boxes covering pore ((**a**) $\mu_{p}=0.0$, D=2.9; (**b**) $\mu_{p}=0.7$, D=2.1).

As illustrated in Figure 10, taking two cases ($\mu_{p}=0.0$, D=2.9 and $\mu_{p}=0.7$, D=2.1) as examples, the log-log plots of the box side ($r_{b}$) and corresponding number of boxes covering pore $N_{b}$ are provided. It can be observed that the linear fitting degrees are very high. The results of the other cases are same as the two cases presented in Figure 10 with a fitting degree of $R^{2}>0.998$, which implies that the pore structures of grain-size fractal granular materials are typical fractal objects.

**Figure 11:**
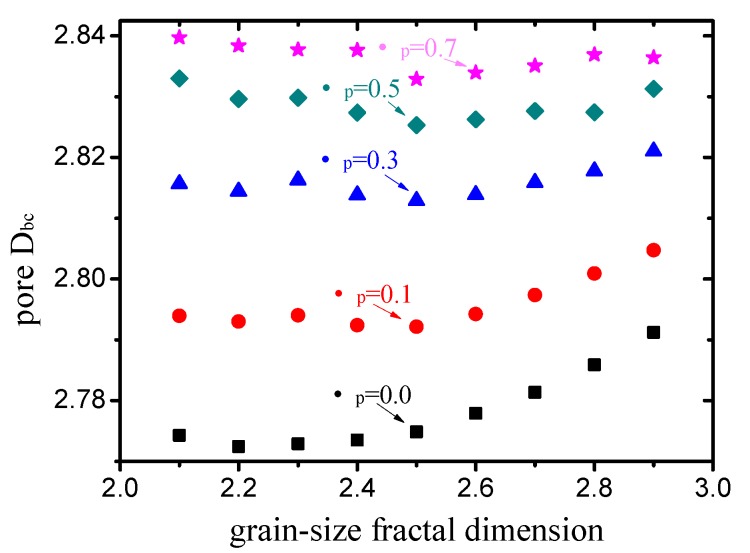
Box-counting dimensions of pore structures with different grain-size fractal dimensions and friction coefficients.

The calculated box-counting dimensions of the pore structures with different grain-size fractal dimensions and friction coefficients (compactness) are illustrated in Figure 11. Overall, the variation in the box-counting dimension of the pore structure is very small. The friction coefficient is more sensitive to the box-counting dimension than the grain-size fractal dimension. When the friction coefficient is small ($\mu_{p}<0.5$), the box-counting dimension tends to increase with an increase in the grain-size fractal dimension. There are no obvious trends of the box-counting dimension with the increase in the grain-size fractal dimension when $\mu_{p}>0.5$.

**Figure 12:**
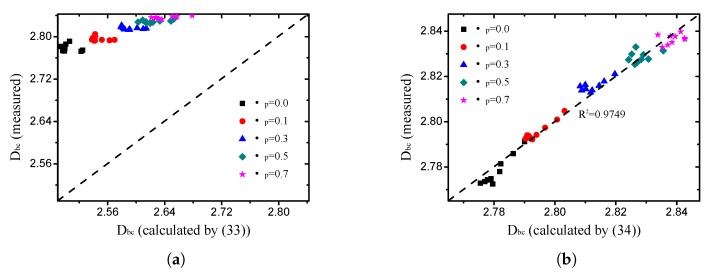
Comparison of box-counting dimensions measured from DEM simulation with values calculated by two different formulae ((**a**) analytic solution (33) of ideal fractal structure; (**b**) empirical relationship (34) provided in the present study).

For an ideal fractal structure (such as the Menger sponge), a certain relationship exists between the porosity and box-counting dimension of the pore, obtained by Yu and Li [35]:

(33) $$D_{\mathrm{bc}}=3-\frac{\ln\phi }{\ln\left(\frac{R_{\min}}{R_{\max}}\right)},$$

in which $R_{\min}$ denotes the minimum pore size and $R_{\max}$ represents the maximum pore size. As illustrated in Figure 12a, (33) is not accurate for the grain-size fractal granular material. The relationship between the fractal dimension and calculated value of (33) is discrete. Therefore, it is impossible to obtain the fractal dimension of the pore from the porosity directly, and other geometric information, such as the grain-size fractal dimension, is needed. In this study, a new relationship, which meets the grain-size fractal granular material, was proposed according to multiple regression:

(34) $$D_{bc}=2.729+0.3049\left(D\phi^{2}\right),$$

It can be observed from Figure 12b that the fitting degree of (34) is very strong, and much better than (33).

#### 3.1.2. Lacunarity

The lacunarity, from the Latin “lacuna” meaning “gap” or “lake,” can reflect the clustering degree (gappiness), heterogeneity, and texture of a fractal structure. Patterns with more or larger gaps generally exhibit higher lacunarity. Beyond providing an intuitive measure of gappiness, it can indicate the differences between structures that have the same or very close fractal dimensions [1].

In this study, the gliding box method described by Allain and Cloitre [68] was used to calculate the lacunarity. A box with a side $r_{b}$ was glided along all possible directions of the binary image of the pore structure. If the sliding box contains *M* points of which the voxel values are 1, the box mass is *M*. The number of boxes with mass *M* is denoted by $n(M,r_{b})$. The probability density function $Q(M,r_{b})$ can be obtained by dividing $n(M,r_{b})$ by the total number of gliding boxes. To analyze the probability density function conveniently, the statistical moments function is constructed as follows:

(35) $$Z_{Q}^{\left(q\right)}\left(r_{b}\right)=\sum_{M}M^{q}Q\left(M,r_{b}\right).$$

The lacunarity can be defined as the statistical moments function of q=2 divided by the square of the statistical moments function of q=1:

(36) $$\Lambda \left(r_{b}\right)=\frac{Z_{Q}^{\left(2\right)}\left(r_{b}\right)}{{\left[Z_{Q}^{\left(1\right)}\left(r_{b}\right)\right]}^{2}}.$$

**Figure 13:**
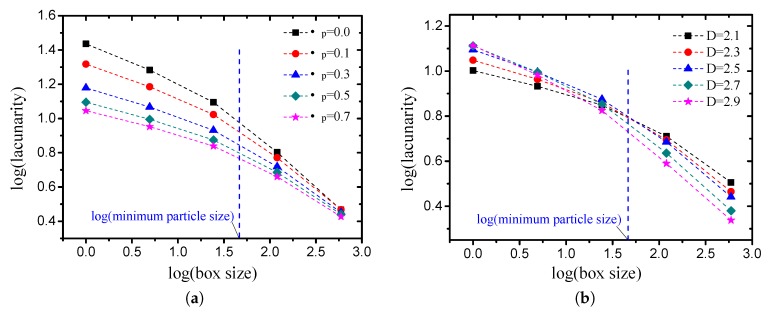
Variation in lacunarity with sliding box size ((**a**) the fractal dimension *D* is 2.5, while the friction coefficient takes different values; (**b**) the friction coefficient $\mu_{p}$ is 0.5, while the fractal dimension takes different values).

The lacunarities of the pore structures with different grain-size fractal dimensions and friction coefficients were calculated by the approach described previously. The calculated lacunarity differs when using different gliding box sizes. The effects of the box size on the lacunarity is illustrated in Figure 13. Figure 13a presents the results of the cases in which the grain-size fractal dimension D=2.5 with different compactness values. Figure 13b illustrates the results of the cases in which the friction coefficient $\mu_{p}=0.5$ with different fractal dimensions. It can be observed from the log-log figures that the curve can be divided into two parts by the minimum particle size, which implies the smallest gap for the pore structure, and the lacunarity decreases linearly with the box size in the two parts with different slopes. With the increase in the gliding box size, the distinction of the lacunarity of the pore structure with different compactness values becomes less obvious. Meanwhile, the distinction of the lacunarity of the pore structure with different fractal dimensions becomes more obvious.

**Figure 14:**
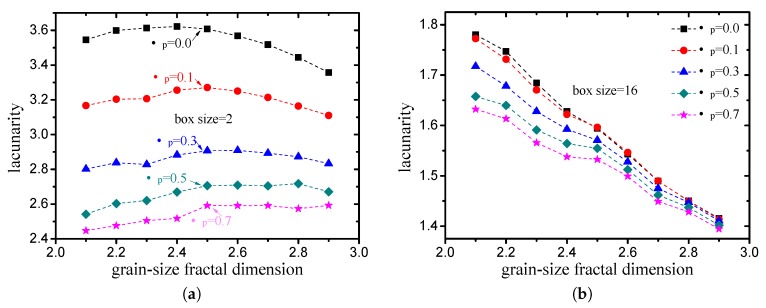
Lacunarity of pore structures with different grain-size fractal dimensions and friction coefficients ((**a**) box size =2 (<minimum particle size); (**b**) box size =16 (>minimum particle size)).

The lacunarity values of the pore structures with different grain-size fractal dimensions and friction coefficients (compactness) are presented in Figure 14. As discussed in Section 3.1.1, the box-counting dimension of each pore structure is very close. It can be observed that the variation in the lacunarity is obvious. When the box size is small (<minimum particle size), the lacunarity can reflect the compactness characteristics effectively, and it decreases obviously with the increase in the friction coefficient (see Figure 14a). When the box size is large (>minimum particle size), the lacunarity can reflect the grain-size gradation characteristics effectively, and it decreases obviously with the increase in the grain-size fractal dimensions (see Figure 14b).

By means of multiple regression, the empirical relationship between the lacunarity (Λ) and porosity (ϕ), and the fractal dimension (*D*) was obtained in this study. When the box size is small (<minimum particle size), the lacunarity is mainly related to the porosity, which can be expressed as:

(37) $$\Lambda \left(r_{\mathrm{bmin}}\right)\propto 1-\frac{1}{\ln(1-\phi )}.$$

**Figure 15:**
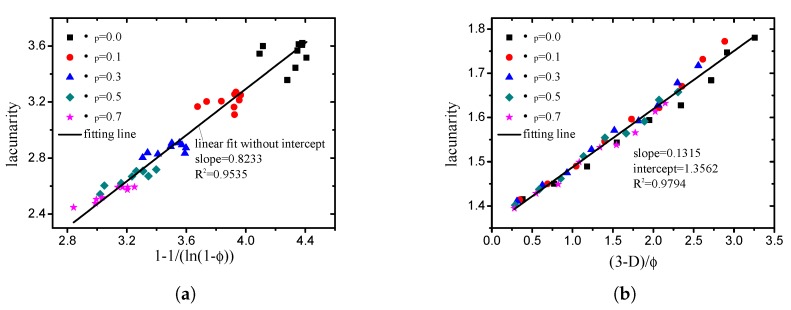
Comparison of lacunarity measured from DEM simulation with values calculated by two empirical relationships provided in this study ((**a**) box size =2 (<minimum particle size); (**b**) box size =16 (>minimum particle size)).

As indicated in Figure 15a, the fitting degree of (37) is 0.9993. When the box size is large (>minimum particle size), the empirical expression of the lacunarity obtained in this study is:

(38) $$\Lambda \left(r_{\mathrm{bmax}}\right)\propto \frac{3-D}{\phi }.$$

The fitting degree of (38) is 0.9794, as illustrated in Figure 15b. Note here that it is difficult to get a box size smaller than the minimum particle size in practice. Therefore, the lacunarity calculated by large box size (>minimum particle size) is more reasonable and important.

In this study, the lacunarity was proven to be a reasonable parameter for distinguishing pore structures with close fractal dimensions. Moreover, the lacunarity can measure the clustering degree of the pore structure. It is generally known that various granular materials are formed by cementation based on particle packing, such as concrete and sedimentary rock. Areas in which the pore concentration of the packing structure before cementation is more pronounced tend to be more vulnerable in these materials. Therefore, it can be stated with certainty that the conclusions regarding lacunarity obtained in this study offer great application potential in the strength theory of these materials.

#### 3.1.3. Succolarity

Succolarity is used to measure the connectivity of the fractal structure in different directions, and can represent the ability of the fluid passing through the medium [69]. In this study, the box counting-based approach proposed by de Melo and Conci [69] was used to calculate the succolarity. The 3D granular media should have six succolarity values in six different directions, owing to the directionality of succolarity. In this study, the particles were arranged randomly in the packing process. Thus, the granular media should be isotropic, and the succolarity should be the same in all directions. The succolarity along the positive direction of *z* was calculated. Briefly, the pore structure was divided into sub-boxes with a side length of *b*. As with the calculation of the lacunarity, the pore coverage ($P_{O}$) was calculated based on the pore mass of each box:

(39) $$P_{O}\left(b,i\right)=\frac{M(i)}{b^{2}}.$$

Then, the “pressure” ($P_{R}$) exerted on each box was stored in an array of pressures. The pressure increases from layer to layer along the positive *z* direction, which can be expressed as

(40) $$P_{R}\left(b,i\right)=\left(0.5+l-1\right)\times b,$$

in which *l* represents the layer of the *i*th box in the *z* positive direction. Finally, the succolarity for the *z* positive direction was calculated by

(41) $$\sigma \left(z+,b\right)=\frac{\sum_{i=1}^{N_{b}}P_{O}\left(b,i\right)\times P_{R}\left(b,i\right)}{{\left(N_{b}\right)}^{2/3}\sum_{i=1}^{N_{b}}P_{R}\left(b,i\right)},$$

where $N_{b}$ is the total number of boxes.

**Figure 16:**
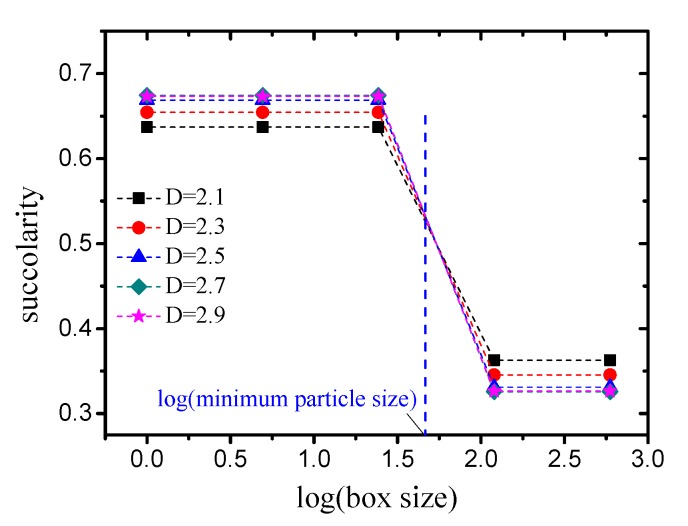
Variation in succolarity with box size (taking D=2.5 as an example).

The effect of the box size on the lacunarity is presented in Figure 16. Similar to lacunarity, the succolarity-log(box size) curve of each case can be divided into two parts according to the minimum particle size. The succoularity takes two different invariant values in each part, and these values are not independent. Therefore, It can be concluded that the succoularity is not affected by the box size.

**Figure 17:**
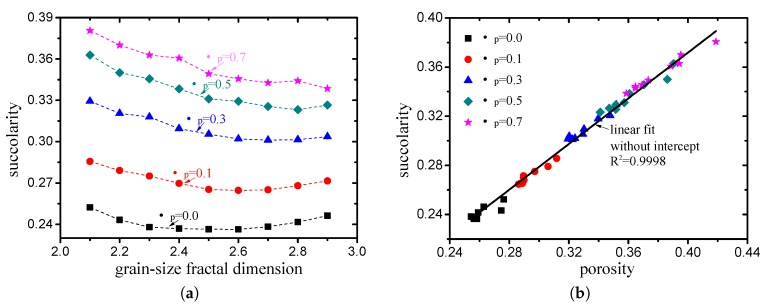
Variation in succolarity with (**a**) grain size fractal dimension and (**b**) porosity.

The succolarity values of pore structures with different grain-size fractal dimensions and friction coefficients (compactness) are presented in Figure 17a (box size takes large value which is larger than the minimum particle size). It can be observed that the variation in the succolarity is highly consistent with the variation in the porosity. Taking the relationship between the succolarity and porosity as

(42) σ∝ϕ,

the fitting degree $R^{2}$ is equal to 0.9998, as indicated in Figure 17b, i.e., a strict linear correlation exists between the succolarity and porosity.

Because the particles used in this study were spherical and the packing was random, the pore structure was isotropic and all of the pore locations were interconnected. Therefore, the ability of the fluid passing through was mainly determined by the porosity, which is why the strict linear correlation appeared.

### 3.2. Pore Size Distribution

#### 3.2.1. Pore Segmentation Method

**Figure 18:**
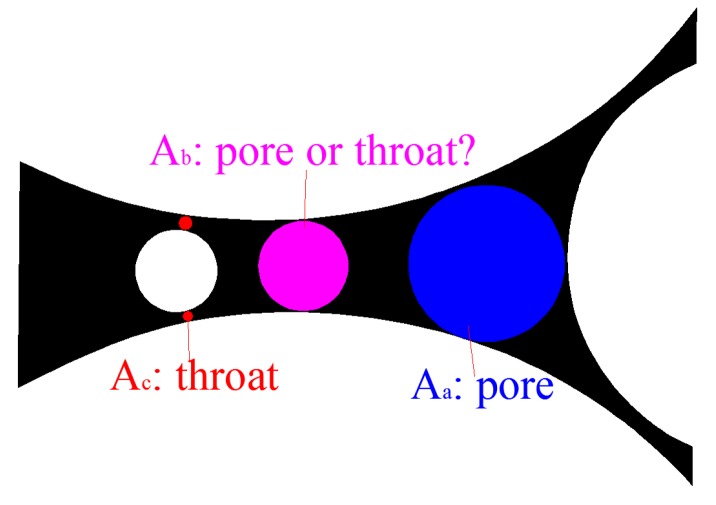
Example of dilemma of distinguishing between pore and throat.

At present, numerous studies exist concentrating on the pore size distribution of the granular material from the pore scale [42,43,44,48,50,53,60]. Several methods have been proposed in these studies, each with its own limitations, as described in Section 1. One of the core issues in these studies is the manner in which to distinguish between pore throats and pores. Different identification criteria can lead to significant variations in the results. In fact, in many cases, it is very difficult to distinguish the throat and pore, particularly for porous media formed by particle packing. For example, as illustrated in Figure 18, there is no doubt that $A_{a}$ is a pore and $A_{c}$ is a throat. However, $A_{b}$ is a throat as it is a constriction to $A_{a}$. Meanwhile, $A_{b}$ is a pore as it is an enlarged part to $A_{c}$. Therefore, it is difficult to define whether $A_{b}$ is a pore or a throat. In many cases, the artificial distinction between a pore and throat makes no sense. Whether for hydraulics or filtration, the movement of fluid or particles in the channel is only sensitive to the size of the cross-section through which they are passing, regardless of whether the size belongs to a pore or throat. Therefore, in many cases, a general segmentation is required, without distinguishing between the pore and throat.

To obtain reasonable segmentation without identifying the pore and throat, a new approach based on continuous morphological open operation was proposed in this study. The mathematical expression for open operation in morphology is

**Figure 19:**
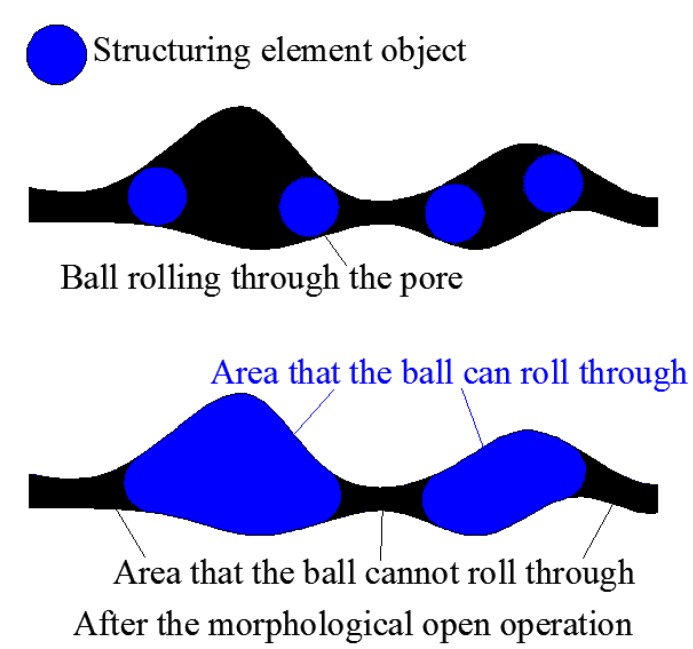
Physical meaning of open operation.

(43) Io=Ii∘Se,

in which Ii is the origin image (the 3D binary image of the pore structure in this study), Se is the structuring element object (a sphere with radius $r_{e}$ in this study), and Io is the binary matrix after open operating. Taking the two-dimensional (2D) structure as an example, herein, the physical meaning of the morphological open operation is described, as illustrated in Figure 19. Taking a disk with a radius $r_{e}$ as the structuring element, the open operation of the pore channel can be regarded as the disk rolling in the channel with the area that the disk cannot roll through being deleted and area that the disk can roll through being retained. As expressed in (43), Io represents the part of Ii through which the structuring ball Se can roll. Let Ia=Ii−Io; then, Ia represents the part of Ii through which Se cannot pass.

**Figure 20:**
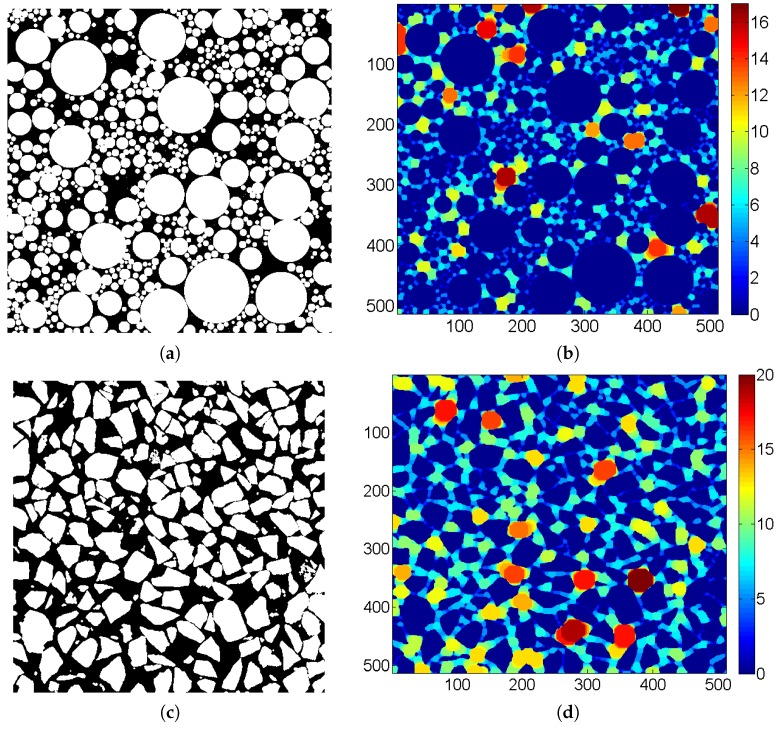
Results of continuous morphological open operation (taking 2D as example) ((**a**) is the origin image and (**b**) is the pore segmentation of the granular media formed by circular particles; (**c**) is the origin image and (**d**) is the pore segmentation of the granular media formed by irregular particles).

By changing the radius $r_{e}$ of the structuring ball from the minimum to maximum, the continuous open operation is applied to the pore structure, recording the radius of the maximum ball that can pass through at each pore structure point. Taking the 2D structure as an example, as illustrated in Figure 20, the distribution contour of the maximum passing ball radius of two types of 2D granular media formed by circular and irregular particles, respectively, are obtained by the continuous morphological open operation. It can be observed that a reasonable segmentation appears after the continuous open operation, following the physical process of filtration. The radius of the maximum ball that can pass through is regarded as the pore size ($r_{p}$) of each point in the pore structure, without identifying the pore and throat. Then, the pore size distribution can be obtained based on the segmentation. The pore segmentation process was implemented by MATLAB programming in this study.

In the introduction to the continuous open operation method described previously, 2D binary images were used as an example, while the pore structure calculated in this study was 3D (see Figure 9). For a 3D binary image, the calculation principle is the same, and only the operation into the 3D open operation needs to be changed, with the 3D sphere as the structuring element. Based on the segmentation methods proposed in this study, the pore size distribution of the pore structures with different grain-size fractal dimensions and friction coefficients were calculated.

#### 3.2.2. Results and Discussion

**Figure 21:**
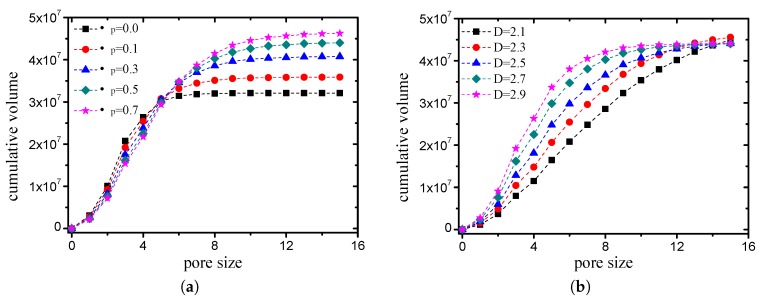
Volume accumulation curves for pore size ((**a**) *D* is 2.5, while the friction coefficient takes different values; (**b**) $\mu_{p}$ is 0.5, while the fractal dimension takes different values).

The volume accumulation curves for the pore size are presented in Figure 21. Figure 21a illustrates the results of the cases in which the grain-size fractal dimension *D* was 2.5 with different compactness values. When the fractal dimensions are the same, the compactness is smaller, while the maximum pore size is larger, i.e., the pore size has a larger distribution range. Figure 21b presents the results of the cases in which the friction coefficient $\mu_{p}$ was 0.5 with different fractal dimensions. When the friction coefficients are the same, the fractal dimension is larger, the content of small pores is larger, and the content of the large pores is smaller. Although the differences between these curves are obvious, the variation law of the curves is consistent, which can be described by a general function.

**Figure 22:**
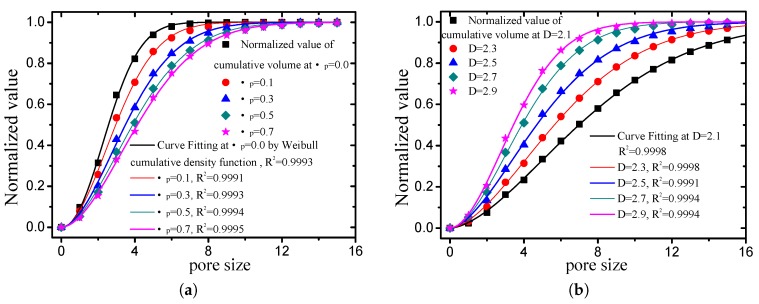
Curve fitting of normalized value of cumulative volume by Weibull cumulative density function ((**a**) *D* is 2.5, while the friction coefficient takes different values; (**b**) $\mu_{p}$ is 0.5, while the fractal dimension takes different values).

In this study, the rule whereby the pore size distribution accords with the well-known two-parameter Weibull distribution [70] was established. The expression of the two-parameter Weibull cumulative function is as follows:

(44) $$F\left(r_{p}\right)=1-e^{-{\left(\frac{r_{p}}{\eta }\right)}^{\lambda }},$$

where $F(r_{p})$ denotes the cumulative volume fraction of the pore size in the $0-r_{p}$ range, η is the distribution scale parameter, and λ is the shape parameter. To determine the rule, the normalized process was applied to the results presented in Figure 21 by first dividing each total pore volume, following which curve fitting was performed on the normalized results using (44). As indicated in Figure 22, the fitting degree of each curve was over 0.999. All other cases not presented in the figure also exhibited a fitness of more than 0.999. Therefore, there is no doubt that the pores of the grain-size fractal granular material following segmentation conform to the two-parameter Weibull distribution.

It must be pointed out that the conclusion of this section is not inconsistent with that of Section 3.1.1, which shows that the pore structures of grain-size fractal granular materials are fractal objects. The box-counting dimension only measures the complexity of the pore structure, but the real pore size distribution is obtained in this section by the pore segmentation method which has strict physical meaning. The curve shape of pore size distribution obtained in this paper is consistent with the theoretical estimation results of Rouault and Assouline [71]. However, Rouault and Assouline [71] just gave the shape of the curves based on some rough theoretical assumptions. In this paper, the pore structure of the particle-packed material is measured directly, and it is proved that the pore distribution of the grain-size fractal granular material satisfies the weibull distribution.

**Figure 23:**
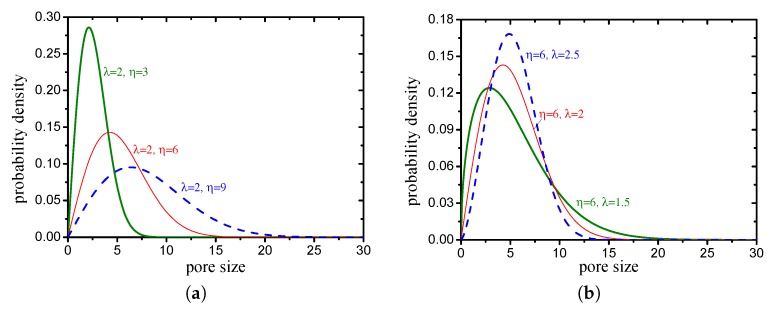
Effects of parameters on Weibull probability density function ((**a**) scale parameter η; (**b**) shape parameter λ).

**Figure 24:**
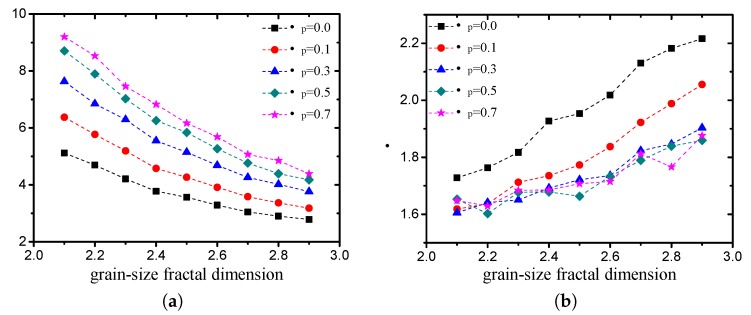
Variation in parameters with fractal dimension and friction coefficient ((**a**) scale parameter η; (**b**) shape parameter λ).

In the Weibull distribution, a change in the scale parameter η has the same effect on the distribution as a change in the abscissa scale (see Figure 23a). In this study, the scale parameter is mainly related to the average pore size. The shape parameter λ is equal to the slope of the regressed line in a probability plot, which determines the shape of the volume probability density function of the pore size (see Figure 23b). The two Weibull parameters of pore structures with different grain-size fractal dimensions and friction coefficients (compactness) are presented in Figure 24. It can be observed that the scale parameter η decreases with an increase in the fractal dimension and increases with an increase in the compactness. The shape parameter λ increases with an increase in the fractal dimension and decreases with an increase in the compactness.

By means of multiple regression, the empirical relationships between the two Weibull parameters (η, λ) and porosity (ϕ), and the fractal dimension (*D*) were obtained:

(45) $$\eta \propto \frac{\phi }{D},$$

(46) $$\lambda \propto e^{\frac{D}{\sqrt{\phi }}}.$$

**Figure 25:**
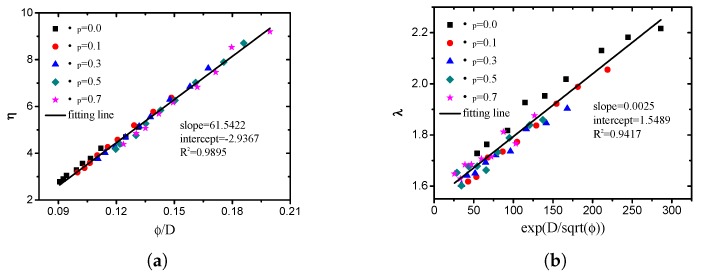
Comparison of values of two parameters obtained from DEM simulation with values calculated by two empirical relationships provided in this study ((**a**) scale parameter η; (**b**) shape parameter λ).

As indicated in Figure 25, the fitting degrees of (45) and (46) were 0.9895 and 0.9417, respectively.

#### 3.2.3. Verification of General Pore Distribution Function

The pore structure of soil has a decisive effect on the soil-water characteristic curve [15,72]. The soil-water characteristic curve is a macro relationship, which can easily be measured in the field or laboratory. However, the real pore structure is difficult to measure. Therefore, an alternative method involves reflecting the pore structure characteristics through the soil-water characteristic curve. To verify the general pore distribution function (44) proposed in this study, a new equation for the soil-water characteristic curve was derived based on (44). Then, the correctness of the new soil-water characteristic equation was verified by comparison with experimental data, which implies that the pore distribution function was verified.

According to the Young-Laplace equation, the relationship between the pore radius $r_{p}$ and capillary pressure ψ is expressed as follows:

(47) $$\psi =C/r_{p},$$

in which $C=2T_{s}\cos\gamma^{*}$, $T_{s}$ denotes the water surface tension, and γ denotes the contact angle. According to the local equilibrium assumption [73], for a specific capillary pressure $\psi^{*}$, pores with sizes greater than $r_{p}^{*}$ in the pore structure are filled with air, while pores with sizes less than $r_{p}^{*}$ are filled with water. At this point, the relative saturation is the percentage of the volume of the pores with sizes less than $r_{p}^{*}$ to the total volume of pores, which is the precise physical meaning of (44). Therefore, based on (44), the relative saturation $S_{e}^{*}$ corresponding to the capillary pressure $\psi^{*}$ can be expressed as

(48) $$S_{e}^{*}=F\left(r_{p}^{*}\right)=1-e^{-{\left(\frac{r_{p}^{*}}{\eta }\right)}^{\lambda }}.$$

Substituting (47) into (48), the new general equation for the soil-water characteristic curve can be derived, which is

(49) $$S_{e}=\frac{\theta -\theta_{r}}{\theta_{s}-\theta_{r}}=1-e^{-{\left(\frac{C}{\eta \psi }\right)}^{\lambda }},$$

where θ denotes the water content corresponding to the capillary pressure ψ, and $\theta_{s}$ and $\theta_{r}$ denote the saturated and residual water contents, respectively.

There are many empirical equations of soil-water characteristic curves, such as van Genuchten equation [17], lognormal-type equation [74] and Weibull-type equation [75,76]. The equation obtained in this paper is similar to the Weibull-type equation proposed by Assouline et al. [75] which can be expressed as follows:

(50) $$S_{e}=1-e^{-\eta {\left(\psi^{-1}-\psi_{\min}^{-1}\right)}^{\lambda }}.$$

There are two basic assumptions in the derivation of Equation (50): the particle size conforms to the Weibull distribution and the pore size is proportional to the particle size. This research does not have these theoretical assumptions, and analysis the pore structure generated by particle packing directly. Furthermore, the physical meaning of pore segmentation in this paper is consistent with that of Young-Laplace equation. Therefore, the calculation in this paper is more accurate than that of Assouline et al. [75]. It can be proved that among these empirical equations, the Weibull-type equation is most reasonable, and the Equation (49) obtained in this paper is simpler than (50).

**Figure 26:**
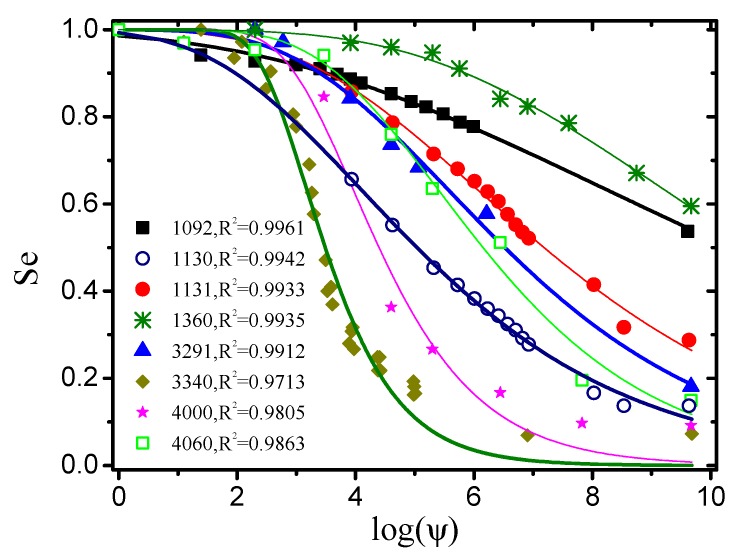
Curve fitting of experimental data from UNSODA by new general soil-water characteristic function derived in this study (numbers such as 1092 represent the sample number in UNSODA).

The experimental dataset for validating the new general Equation (49) of the soil-water characteristic curve was obtained from the UNsaturated SOil hydraulic property DAtabase (UNSODA) [77]. UNSODA is a database of unsaturated soil hydraulic properties (water retention, hydraulic conductivity, and soil water diffusivity), basic soil properties (particle-size distribution, bulk density, organic matter content, etc.), and additional information regarding the soil and the experimental procedures. There are 790 soil samples from different regions of the world in UNSODA. Eight sets of cases with relatively complete experimental data were randomly selected from the UNSODA. Curve fitting was applied to the drying branch of the θ−ψ data points of these cases by the function (49). It can be observed from Figure 26 that the fitting degrees were all above 0.97, which indicates that (49) is a reasonable and general function for expressing the soil-water characteristic curves. As (49) was derived on the basis of (44), it can be stated that the correctness and generality of (44) were also verified.

## 4. Conclusions

Grain-size fractal granular materials are common in natural environments, such as general soil, sedimentary rock, and concrete. It is inappropriate to treat these stacked materials as ideal fractal structures. In this study, the general rules of the pore structures of grain-size fractal materials were explored by means of theoretical analysis, DEM simulation, and digital image processing. Several important conclusions were obtained, as follows:

1. Based on bi-dispersed packing and the geometric packing theory, the relationship between the porosity and fractal dimension of grain-size fractal granular material was established, which can be used to predict the porosity. The rationality of the relationship was verified by means of simulation of the packing processes under the conditions of different fractal dimensions and compactness values using DEM.
2. The fractal properties were studied based on the 3D binary image of the pore structure extracted from the DEM packing simulation. The pore structure of grain-size fractal granular material conforms to the fractal law, but the box-counting dimension differs from the ideal fractal structure, such as the Menger sponge. The empirical relationship between the box-counting dimensions, lacunarity, succolarity, and grain-size fractal dimension, and the porosity were provided in this study.
3. Whether for hydraulics or filtration, the movement of fluid or particles in the channel is only sensitive to the size of the cross-section through which they are passing, regardless of whether the size belongs to a pore or throat. A new segmentation method for the pore structure without distinguishing between the pore and throat was proposed based on the continuous morphological open operation. According to the segmentation results, a general function of the pore size distribution was established, of which the generality and correctness were verified by the soil-water characteristic curves from the experimental database.

These conclusions were obtained by continuously graded materials and they cannot be applied to gap-graded materials. Any materials can use these conclusions as long as they satisfy the following conditions: (1) The materials are formed by particle packing; (2) The size distribution of particles satisfies the number-size fractals law.
